# Vaccination Against SARS-CoV-2 in Immunosuppressed Patients with Rheumatic Diseases: Position Statement of the Greek Rheumatology Society

**DOI:** 10.31138/mjr.31.4.430

**Published:** 2020-12-28

**Authors:** The Greek Rheumatology Society and Professional Association of Rheumatologists (ERE-EPERE)

**Keywords:** Position statement, vaccination, SARS-CoV-2

## INTRODUCTION

Patients with autoimmune inflammatory rheumatic diseases are at higher risk for serious complications of bacterial or viral infections that can be prevented by appropriate vaccination.^1–3^ The European League Against Rheumatism (EULAR)^2^ and the Greek Rheumatology Society and Professional Association of Rheumatologists (ERE-EPERE)^4^ have published their recommendations regarding vaccination in this population.

Considering the upcoming vaccination of the population worldwide against SARS-CoV-2, the Greek Rheumatology Society presents the available data and Recommendations from international^5–9^ and local^10^ Health Authorities as well as its Position regarding the vaccination of immunosuppressed rheumatic patients.

## WHICH ARE THE AVAILABLE VACCINES AGAINST SARS-CoV-2 TODAY?

To date (December 2020), 5 vaccines against SARS-CoV-2 have completed phase 3 studies, while more than 50 are in various phases of clinical development. One vaccine (Pfizer BioNTech COVID-19) has been approved for emergency use by the regulatory authorities in the United Kingdom (UK), the United States (US, Food and Drug Administration-FDA), and in European countries (European Medicines Agency-EMA). So far, vaccination has already started in the UK and US. Another one (Moderna COVID-19) has just received emergency use authorization from the US FDA. Both of these vaccines are nucleoside-modified messenger RNA (mRNA) vaccines.

## HOW ARE THE mRNA VACCINES BEING ADMINISTERED, AND HOW DO THEY WORK?

Both vaccines are administered intramuscularly in two doses, 3 (Pfizer BioNTech COVID-19) or 4 (Moderna COVID-19) weeks apart. Both contain modified mRNA packed in lipid nanoparticles which after vaccination is incorporated into human cells and leads to the production of the viral spike glycoprotein S.^10–12^ Such vaccines are highly immunogenic in humans eliciting strong B cell (production of protective antibodies) and T cell responses,^11,13–16^ although the duration of their protection remains to be determined.

Since these vaccines do not contain the live virus, there is no danger for viral transmission after vaccination, while mRNA is destroyed after a few days by the host.^11^

## WHAT IS THE EFFICACY AND SAFETY OF THE NEW mRNA VACCINES?

Available data from human randomised controlled trials (RCTs) to date for these two mRNA vaccines show that their efficacy for preventing symptomatic COVID-19 infection ranges from 94%^16^ to 95%,^11,17^ while their respective efficacy in preventing severe COVID-19 infection ranges from 89%^11,18^ to 100%.^17^

Regarding safety, the short-term safety data available, show that vaccinated persons experience mostly local (pain, erythema) or mild systemic reactions (fatigue, headache, fever, arthralgias).^6,12,17–20^ These mild side-effects are similar to those reported from other vaccines used in clinical practice for decades.

Severe anaphylactic reactions have not been observed in the clinical trials of both vaccines, but there have been rare cases of anaphylaxis in persons vaccinated outside of clinical trials as with any other vaccine. These vaccines will be administered worldwide in COVID-19 vaccination centres under direct medical supervision and observation.^6^

## WHAT ARE THE CONTRA-INDICATIONS TO mRNA VACCINE ADMINISTRATION?

According to their authorisation use prescribing information, these vaccines are only contra-indicated in individuals with a known history of severe allergic reaction (eg, anaphylaxis) to any of their components.^6,19,20^ Patients with such history should discuss it prior to their vaccination with the health personnel of the COVID-19 vaccination centres.

## WHAT IS THE SAFETY AND EFFICACY OF THE mRNA VACCINES IN IMMUNOCOMPROMISED PATIENTS WITH RHEUMATIC DISEASES?

Data regarding the safety and efficacy of these vaccines in immunocompromised patients is at present limited. Although, in the clinical studies that led to the approval of these vaccines immunocompromised patients were excluded,^12,17^ in one of the 2 RCTs, patients with history of malignancy (n=1.395), AIDS/HIV (n=121) or rheumatic disease (n=118) were included.^12^ There was no difference in the vaccine efficacy or safety in these patients compared to the rest of the vaccinated patients.

Nevertheless, patients with rheumatic conditions who are immunocompromised due to their immunosuppressive therapy or due to their disease may have a decreased response to these new vaccines as with other vaccines currently in clinical use.^2,3^

## SHOULD IMMUNOCOMPROMISED PATIENTS BE VACCINATED WITH THE APPROVED VACCINES AGAINST SARS-CoV-2?

Health authorities in the UK,^7^ US,^5–6^ and other countries including Greece^10^ recommend that all immunosuppressed patients should be vaccinated against SARS-CoV-2.

In general, in these early phases of the vaccination process where the availability of the vaccines may be limited, priority for vaccination has been given to critical infrastructure workers (such as frontline health care personnel, staff working in nursing homes, etc.) and high-risk patients for severe COVID-19 (including elderly people, residents of nursing homes and assisted living facilities, as well as patients with underlying medical conditions, which put them at higher risk for severe disease or death).^5–7^

Patients who are immunosuppressed due to their disease or immunosuppressive therapies are generally included in the prioritized high-risk population for vaccination against SARS-CoV-2.^5–7^

## POSITION STATEMENT

Based on the long-term experience with vaccinations in patients with rheumatic diseases,^1–3^ the available data from clinical trials,^12,17^ and the most recent recommendations from Health Authorities,^5–7,10^ the Greek Rheumatology Society (ERE-EPERE) recommends that all immunosuppressed patients with rheumatic conditions should be vaccinated against SARS-CoV-2.

The prioritisation of immunosuppressed patients for vaccination will be determined by the Health Authorities. We also recommend that patients should always contact their rheumatologist with any questions that may have prior to their vaccination.

Vaccinated patients should continue following existing guidelines regarding protective measures for themselves and people around them (wearing masks, keeping distance from others, avoiding crowds, etc.).

This statement will be updated as more data regarding the safety and efficacy of these and other vaccines become available.
